# Effectiveness and Safety of Bariatric Surgery in the Public Healthcare System in Brazil: Real-World Evidence from a High-Volume Obesity Surgery Center

**DOI:** 10.1007/s11695-016-2439-y

**Published:** 2016-11-25

**Authors:** Irineu Rasera, Alexandre Luque, Silvio Mauro Junqueira, Níssia Capello Brasil, Priscila Caldeira Andrade

**Affiliations:** 1 Bariatric Surgery, Clínica Bariátrica, Hospital dos Fornecedores de Cana de Piracicaba, Piracicaba, Brazil; 2 HEMA Brazil, Johnson & Johnson Medical Devices Brazil, Rua gerivatiba, 207, 12 andar, Butantã, São Paulo, SP Brazil; 3 Medical Affairs/Assuntos Médicos, Johnson & Johnson do Brasil Ind. e Com. de Produtos para Saúde Ltda., Rod. Presidente Dutra, km 154, São José dos Campos, SP 12240-908 Brazil

**Keywords:** Open Roux-en-Y gastric bypass, Real-world evidence, Public health, Diabetes resolution, Bariatric surgery

## Abstract

**Background:**

This study aimed to evaluate the waiting time, safety, and effectiveness of bariatric surgery based on real-world data.

**Methods:**

This is a noninterventional, noncomparative, and retrospective study with 300 morbidly obese patients who had undergone open Roux-en-Y surgery.

**Results:**

The procedure was found to be very safe, with low rates of overall complications (10.7%). Approximately 48.4% of the patients had reached a BMI <30 mg/kg^2^ at 12 months after surgery, while 6% were still classified as morbidly obese (BMI >40 mg/kg^2^). Comorbidity resolution was over 90% for all conditions, except for cardiovascular disease, which showed a 40% resolution. The mean number of drugs taken also decreased at 12 months after surgery.

**Conclusions:**

Bariatric surgery was found to be effective in weight reduction and in the resolution of comorbidities.

## Introduction

Obese people have a higher risk of type 2 diabetes, cardiovascular diseases, hypertension, and several types of cancer, and the higher prevalence of comorbidities is directly connected with increased medical spending. Brazilian health authorities estimate a prevalence of 16.8% for obesity within the Brazilian population. Therefore, an effective approach to manage obesity should be implemented, and bariatric surgery has proven to be a therapeutic option for long-term weight control in morbidly obese patients [1–5].

Brazilian health authorities have included bariatric surgery (open Roux-en-Y gastric bypass) as a public, reimbursable procedure since 2001. Procedure outcomes is available on the National Database for the public healthcare system records, but information regarding waiting time, long-term safety, comorbidity resolution, weight loss, and number of pharmaceutical prescriptions per patient is not available. This limits the ability to evaluate the safety and relevant clinical outcomes in Brazil with real-world data. This is especially important given that globally, most real-world data on bariatric surgery is on laparoscopic procedures, while in Brazilian public system, it is performed as an open procedure.

This study aimed to evaluate the waiting time, safety, and effectiveness of bariatric surgery, in addition to comorbidity resolution, weight loss, and drugs taken, based on real-world data from the third highest volume bariatric surgical center within the Brazilian public system.

## Materials and Methods

This is a noninterventional, noncomparative, and retrospective study. The study population consisted of 300 morbidly obese patients who underwent open Roux-en-Y surgery, performed between January 1, 2010, and December 31, 2010. The procedures were performed in the same center and by the same surgical team, in order to standardize differences among surgeons’ skills and preoperative and postoperative patients’ care. It is noteworthy that the center where patients were recruited from performed nearly 700 operations in 2015, which represents roughly 10% of the total volume in the public system. Main inclusion criteria were consistent with eligibility criteria described in the Brazilian Public Health Guidelines at the time the study was conducted: age 18–65 years old, BMI ≥40 kg/m^2^ (not responding to conservative treatment for at least 2 years or a life-threatening condition resulting from comorbidities) or BMI ≥35 mg/m^2^ in combination with at least one severe comorbidity associated with obesity (hypertension, diabetes, etc.) [6], and at least 1 year postprocedure follow-up data. In addition, open Roux-en-Y should be the primary intended and indicated procedure. Patients were excluded if they had any other clinically significant condition not related to obesity (advanced cancer, HIV, viral hepatitis, drug abuse, car accident) or presented any condition that could impact on study results, such as pregnancy or participation in other study. Patient data was collected in the period of time from baseline (visit prior to surgery) until 12 months after surgery. The 300 patients recruited for this study in 2010 represent 75% of all Roux-en-Y surgeries performed in the selected center within that year; the other 25% were not selected based on exclusion criteria. Comorbidity resolution was defined according to surgical center criteria: patients achieving normal range values of target biomarkers (HbA1c, blood pressure, and lipid levels) without the need of pharmacotherapy. Waiting time was defined as the period when surgery was indicated for the patient until the effective surgical date. Data was registered in an electronic formulary and externally and systematically monitored.

Demographic and baseline clinical characteristics and efficacy endpoints were described using the mean and standard deviation for continuous measures (e.g., age, BMI) and the frequency and percentage for categorical variables (e.g., sex, comorbidities).

### Statistical Analysis

Comparison of weight loss, BMI, and excess weight after 12 months of follow-up and baseline was performed with a paired *t* test. A multivariate analysis was developed to estimate the effect of surgery on individual patient’s weight loss, and the potential predictor variables in the model included demographic variables and baseline comorbidities. Point estimates and exact confidence intervals were calculated for comorbidity resolution for diabetes, hypertension, sleep apnea, dyslipidemia, and cardiovascular disease during the follow-up period. Tabulation of summary statistics, graphical presentations, and data analysis were performed using SAS software version 9.2 (SAS Institute, Cary, NC).

## Results

Baseline patient characteristics are summarized in Table 1, and among study subjects, the time on the wait list ranged from 1.0 to 94.0 months. During this period, patients had an average BMI reduction of 2.7 ± 2.7 kg/m^2^ and 37 (12%) patients gained weight while waiting for surgery (4.96 ± 3.70 kg). In addition, while on the wait list, the median number of physician visits was 8, which represents approximately one visit every 3 months.

**Table 1:** Baseline characteristics

Category	
Age (years), mean ± SD	37.8 ± 9.33
Gender (female), *n* (%)	270 (90%)
Baseline BMI (kg/m^2^), mean ± SD	46.5 ± 7.57
Comorbidity	
Diabetes, *n* (%)	55 (18.3%)
Hypertension, *n* (%)	195 (65.0%)
Sleep apnea, *n* (%)	80 (26.7%)
Dyslipidemia, *n* (%)	70 (23.3%)
Cardiovascular disease, *n* (%)	10 (3.3%)
Gastric bypass, *n* (%)	300 (100%)
Gastric bypass with silastic ring, *n* (%)	52 (17.3%)
Time on the wait list (months), mean ± SD	24.7 ± 10.6

After the surgery, none of the patients needed to be admitted to the intensive care unit and the patients were discharged within 3 days on average. Total procedure-related complication rates were low (10.0%) (Table 2). No patient died during the study period.

**Table 2:** Incidence of procedure-related complications in patients undergoing Roux-en-Y gastric bypass

Type of complication	Number of patients (%)
Major complication	
Seven-day hospital readmission	2 (0.7)
Intestinal obstruction	9 (3.0)
Migration of gastric band	3 (1.0)
Severe surgical site infection	3 (1.0)
Digestive hemorrhage with blood transfusion	1 (0.3)
Anastomotic ulcer or gastric stump	2 (0.7)
Severe post-op depression	1 (0.3)
Minor or late complications	
Incisional hernia	9 (3.0)
Gastric foreign body	5 (1.7)
Other	3 (1.0)

Six patients presented 2+ complications

**Fig. 1:**
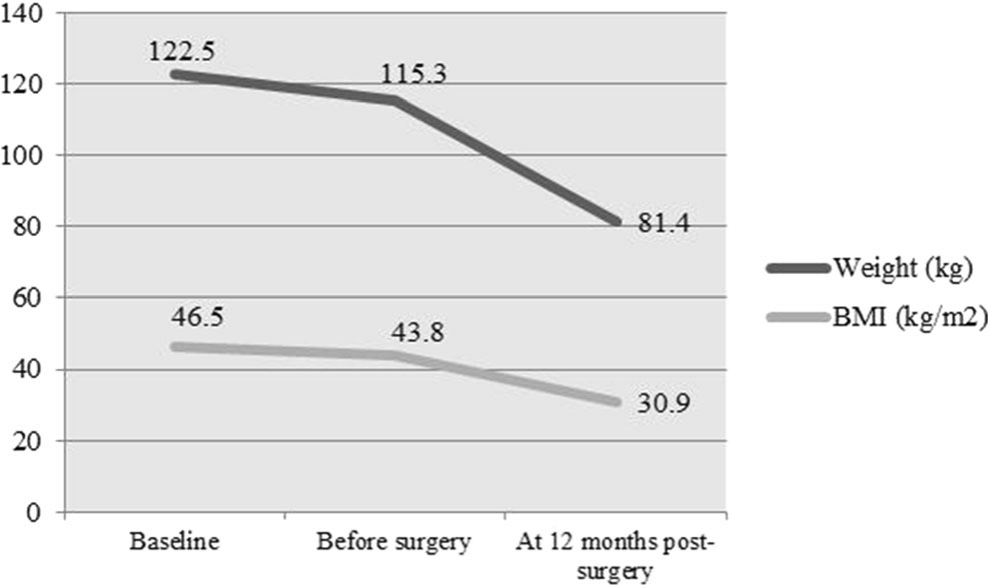
Change on weight and body mass index (BMI) over time percent of weight and BMI loss: baseline–before surgery = 5.7%; baseline–at 12 months postsurgery = 33.4%

In terms of weight loss, the patients on average experienced a 33.4% reduction of their total BMI (Table 3 and Fig. 1). Approximately 48.4% of the patients had reached a BMI <30 mg/kg^2^, and 6% were still classified as morbidly obese (BMI >40 mg/kg^2^) at 12 months after surgery (Table 4).

**Table 3:** Weight change and body mass index (BMI) in patients undergoing to bariatric surgery

Parameter	Baseline	12 months after surgery	Δ	*p* value*
Weight (kg)	122.5 (22.96)	81.4 (16.36)	41.1 (12.17)	0.001
BMI (kg/m^2^)	46.5 (7.57)	30.9 (5.54)	15.6 (4.38)	0.001
Excess weight (kg)	56.93 (20.39)	15.79 (14.48)	41.14	0.001

**Table 4:** Proportion of patients per body mass index (BMI)

	Baseline		12 months after surgery	
Category	*n*	%	*n*	%
BMI <25	0	0.0	57	12.7
BMI 25–29.9	0	0.0	115	35.7
BMI 30–34.9	3	1.0	74	29.3
BMI 35–39.9	48	16.0	37	16.0
BMI 40–44.9	102	34.0	13	4.0
BMI 45–49.9	65	21.7	4	1.7
BMI ≥50	82	27.3	0	0.3

### Comorbidity Resolution

**Fig. 2:**
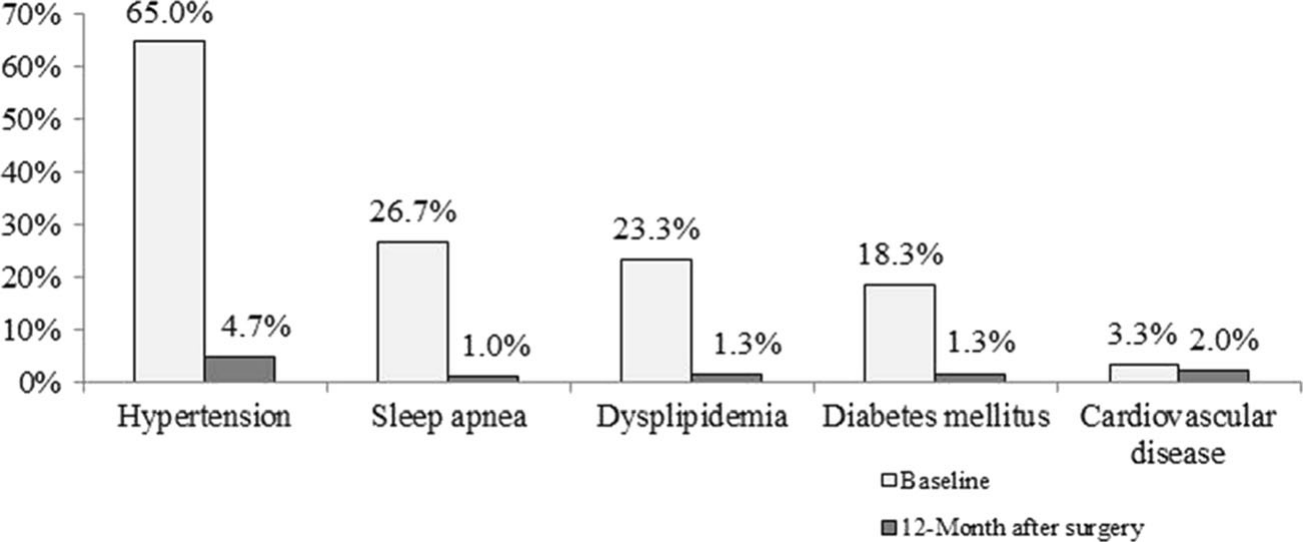
Proportion of patients with obese-related comorbidities

At baseline, 235 patients (78%) had at least one obesity-related comorbid condition, hypertension being the most common (65%) (Fig. 2). Comorbidity resolution was over 90% for all conditions, except for cardiovascular disease, which showed a 40% resolution (Table 5). The mean number of drugs taken increased from 1.35 ± 1.58 to 2.3 ± 1.56 (*p* < 0.01) while the patients were on the wait list and then decreased to 1.2 ± 0.7 at 12 months after surgery, the therapeutic class of drugs taken are described in Table 6. All patients received polyvitamins after the surgery.

**Table 5:** Estimates of comorbidity resolution

Comorbid condition	Follow-up (months)	Estimate	95% Confidence limits^a^		Sample size
Diabetes mellitus	12	0.93	0.82	0.98	55
Hypertension	12	0.93	0.88	0.96	195
Sleep apnea	12	0.96	0.89	0.99	80
Dyslipidemia	12	0.96	0.88	0.99	70
Cardiovascular disease	12	0.40	0.12	0.74	10

^a^Clopper-Pearson (exact) confidence limits

**Table 6:** Percentage of therapeutic classes within medications taken

Drug class	Basal (%)	At surgery (%)	12 months after surgery (%)
Antihypertensive	66.2	59.1	1.4
Oral antihyperglycemic	12.3	12.1	1.4
Insulin	3.7	3.7	0
Lipid-lowering	3.1	3.0	0.0
Anxiety control	1.5	1.5	0.0
Antidepressants	3.1	4.5	1.4
Hypothyroidism	7.7	7.6	7.0
Anemia	0.0	0.0	1.4
Anticoagulant	1.5	1.5	0.0
Inhaled corticosteroids	1.5	1.5	1.4

## Discussion

Randomized clinical trials have strict and specific inclusion/exclusion criteria, in addition to more frequent follow-up visits and exams. In comparison, our study aimed to evaluate the clinical impact of bariatric surgery using real-world data of patients undergoing surgery in the Brazilian public healthcare system.

In this study, bariatric surgery (open Roux-en-Y gastric bypass) had a low procedure-related complication rate (10.0%) This is reflected by the short length of hospitalization (3 days) and by the fact that no patient was admitted to the ICU after the procedure. Paxton et al. performed a cost-effectiveness study comparing data from laparoscopic versus open gastric bypass surgery. In their study, procedure complications were a key parameter and the meta-analysis they performed showed that patients who had undergone open surgery were more likely to experience major extraintestinal effects, fistula formation, wound infection, and ventral incisional hernia [7].

In our study, the mean percent reduction for absolute weight and BMI was around 30%. At 12 months after surgery, 48.4% had a BMI <30 kg/m^2^, and 6% were still considered to be morbidly obese. However, it is noteworthy that these patients had extremely high BMI at baseline (50–80 kg/m^2^).

Several programs recommend that patients lose 5–10% of total body weight prior to surgery, which has been associated with improved surgical outcomes and greater postoperative weight loss. The mean interval between indication for surgery and the procedure was long (over 2 years) and the patients experienced 5.7% total body weight loss during this period compared with basal weight or 17.58% of total body weight loss (TBWL). It has been demonstrated that the patients may benefit from close monitoring of weight preoperatively; therefore, our program performs periodic visits while on the wait list, on average one visit quarterly [8].

The resolution of comorbidities is a desired outcome, and in some cases, may be the main reason for treating morbidly obese patients. At 12 months after surgery, 208 patients (who had at least one comorbidity at baseline) experienced resolution of their comorbid condition. Estimates of comorbidity resolution were over 90% for hypertension, diabetes mellitus, sleep apnea, and dyslipidemia. This is reflected by the number of concomitant drugs taken by the patients. Patients at baseline used an average of 1.35 drugs, with an increase to 2.3 drugs while on the wait list, indicating a possible deterioration of their condition. The number of average number of drugs taken by a patient decreased to 1.2 drugs at 12 months after surgery.

These results are consistent with those observed from other Brazilian and international studies. Pinhel et al. conducted a retrospective study on data of 598 Brazilian morbidly obese patients (grade III, >40 mg/kg^2^), aiming to evaluate the impact of bariatric surgery solely on weight loss and metabolic outcomes. They also found that their study population experienced weight loss and significant reduction in the proportion of patients with diabetes mellitus and dyslipidemia; however, their study did not include any safety data [2].

Additionally, Chang et al. conducted a systematic review with 259 articles. Similarities were seen in baseline characteristics (higher proportion of female and white patients, mean age of 45 years, and BMI of 46). They found a complication rate of 17%. BMI changes of approximately −12 kg/m^2^ at year 1 were observed, and comorbid conditions significantly improved after surgery [3].

Obesity and associated diseases have a significant financial impact on the public health system. In 2011, the cost burden of obesity and related conditions was estimated at US$269.6 million, which represents 1.86% of the budget for medium- and high-complexity healthcare expenses in the Brazilian public health system. Morbid obesity costs totaled $64.2 million, and the highest costs were attributable to diabetes and ischemic heart disease. Bariatric surgery is also included in the total costs associated with morbid obesity, and bariatric surgical costs are estimated at $17.4 million. An effective approach causing the improvement or resolution of comorbidities may lead to a significant reduction of concomitant therapy and therefore, reduce total treatment costs for morbidly obese patients [4, 9].

## Conclusion

The inclusion of bariatric surgery as a reimbursable procedure by Public Health was an advance in the treatment of morbidly obese patients. Our “real-world” experience in an accredited center demonstrated beneficial outcomes with respect to weight loss and the resolution of obesity-related comorbidities. Health authorities should develop and implement programs to stimulate high-volume centers to replicate such results across the country.
